# You Dropped the Bomb on Me: A Case Series of Carbon Tetrachloride Toxicity

**DOI:** 10.7759/cureus.37879

**Published:** 2023-04-20

**Authors:** Amanda R Stashin, Derek J Fikse, Armando M Orta, Robert P Briggs, Scott M Wheatley, Andrew L Koons

**Affiliations:** 1 Department of Emergency and Hospital Medicine, Lehigh Valley Health Network, University of South Florida Morsani College of Medicine, Allentown, USA; 2 Division of Medical Toxicology, Department of Emergency and Hospital Medicine, Lehigh Valley Health Network, University of South Florida Morsani College of Medicine, Allentown, USA; 3 Department of Emergency and Hospital Medicine, Lehigh Valley Health Network/ University of South Florida Morsani College of Medicine, Allentown, USA; 4 Division of Critical Care Medicine, Department of Pediatric Critical Care, Lehigh Valley Health Network, University of South Florida Morsani College of Medicine, Allentown, USA; 5 Division of Medical Toxicology, Department of Emergency Medicine, Lehigh Valley Health Network, University of South Florida Morsani College of Medicine, Allentown, USA

**Keywords:** n-acetylcysteine, hepatocellular necrosis, hepatotoxin, severe acute liver toxicity, carbon tetrachloride

## Abstract

Carbon tetrachloride (CCl_4_) is a halogenated hydrocarbon that is a colorless, clear liquid with a sweetish, ether-like, nonirritant odor. It was previously used in dry cleaning agents, refrigerants, and fire extinguishers. CCl_4_ toxicity is rarely observed. Two patients with acute hepatitis following exposure to a CCl_4_-containing antique fire extinguisher are presented. A son (patient 1) and father (patient 2) were admitted to the hospital with acute, unexplained elevated transaminases. After extensive questioning, they reported recent exposure to a large amount of CCl_4_ when an antique *firebomb* shattered in their home. Both patients cleaned the debris without personal protective equipment and slept in the contaminated area. The patients presented to the emergency department (ED) at varying times between 24 and 72 hours after CCl_4_ exposure. Both patients received intravenous *N*-acetylcysteine (NAC); patient 1 also received oral cimetidine. Both recovered uneventfully without sequelae. Extensive workup for other causes of elevated transaminases was unremarkable. Serum analyses for CCl_4_ were also unremarkable due to the delay between exposure and hospital presentation. CCl_4_ is a potent hepatotoxin. CCl_4_ metabolism via cytochrome CYP2E1 produces its toxic metabolite, the trichloromethyl radical. This radical covalently binds to hepatocyte macromolecules and causes lipid peroxidation and oxidative damage with ensuing centrilobular necrosis. Treatment is not well established, but NAC is likely beneficial via glutathione repletion and antioxidant effects. Cimetidine blocks cytochrome P450 and, thus, metabolite formation. Cimetidine may also promote the stimulation of regenerative processes acting on DNA synthesis. CCl_4_ toxicity is rare and infrequently reported in current literature but should be maintained in the differential of acute hepatitis. Two patients presenting nearly identically - at two different ages but from the same household - offered a clue to this enigmatic diagnosis.

## Introduction

Carbon tetrachloride (CCl_4_, tetrachloromethane) is a halogenated hydrocarbon that is a colorless, clear liquid with a sweetish, ether-like, nonirritant odor. it was previously used in dry cleaning agents, refrigerants, and fire extinguishers [1]. Currently, its commercial use is banned, but it can still be found as an intermediate in chemical manufacturing [2]. CCl_4_ is most recognized as a potent hepatotoxin due to its metabolism to trichloromethyl radical [3]. CCl_4_ toxicity is rarely observed and typically only after repeated exposure in an occupational setting. We present a case series of two patients with severe acute liver toxicity following a single exposure to CCl_4_ from an antique fire extinguisher.

This paper was previously presented as an abstract at the 2022 Pennsylvania College of Emergency Physicians Scientific Assembly on April 1, 2022, and the 2022 North American Congress of Clinical Toxicology on September 16, 2022.

## Case presentation

Patient 1

A 17-year-old male with type 1 diabetes presented to the emergency department (ED) with nausea, vomiting, abdominal pain, and hyperglycemia. His initial vital signs (VS) were as follows: blood pressure (BP), 121/73 mmHg; heart rate (HR), 115 beats per minute; temperature (T), 98.9 °F; respiratory rate (RR), 30 breaths per minute; and oxygen saturation (SpO2), 100% on room air. He was ill-appearing, with dry mucous membranes and diffuse abdominal tenderness. He was found to be in diabetic ketoacidosis (DKA) with pH 7.21 (7.35-7.45), bicarbonate 11 mg/dL (23-31), anion gap 19 (3-11), and glucose 445 mg/dL (65-99). He also had significantly elevated transaminases with aspartate aminotransferase (AST) 2,819 U/L (<41) and alanine aminotransferase (ALT) 2,344 U/L (<56). Total bilirubin was 1.9 mg/dL (0.1-0.8), prothrombin time (PT) 18.9 seconds (11-13.5), and international normalized ratio (INR) 1.7 (0.8-1.1). Imaging of the abdomen had unremarkable findings. The patient’s DKA rapidly resolved following treatment per standard protocol.

**Figure 1:**
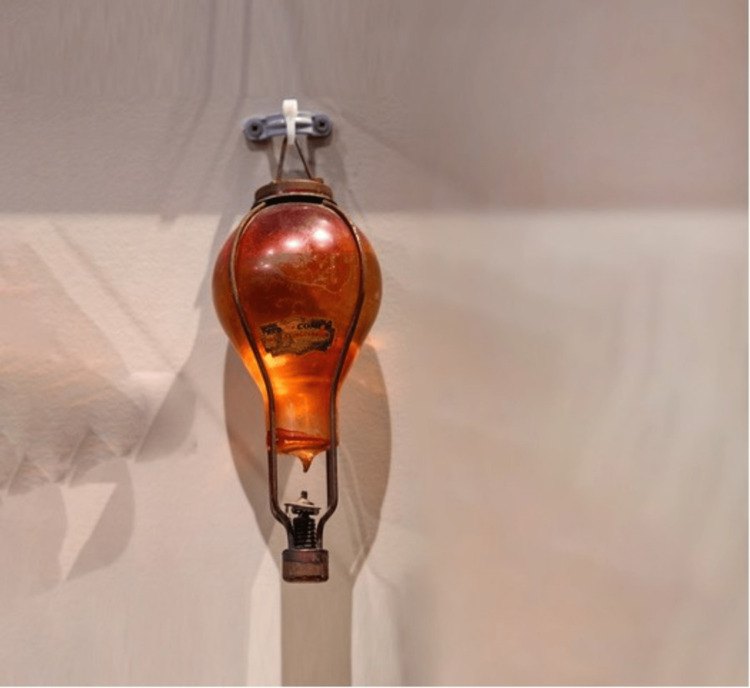
An antique firebomb. Kearney, Nebraska, USA - 12/2019: Glass Grenade Style Fire Extinguisher filled with carbon tetrachloride at the Nebraska Fire Museum for editorial use only. Photo credit: Lost_in_the_Midwest

**Figure 2:**
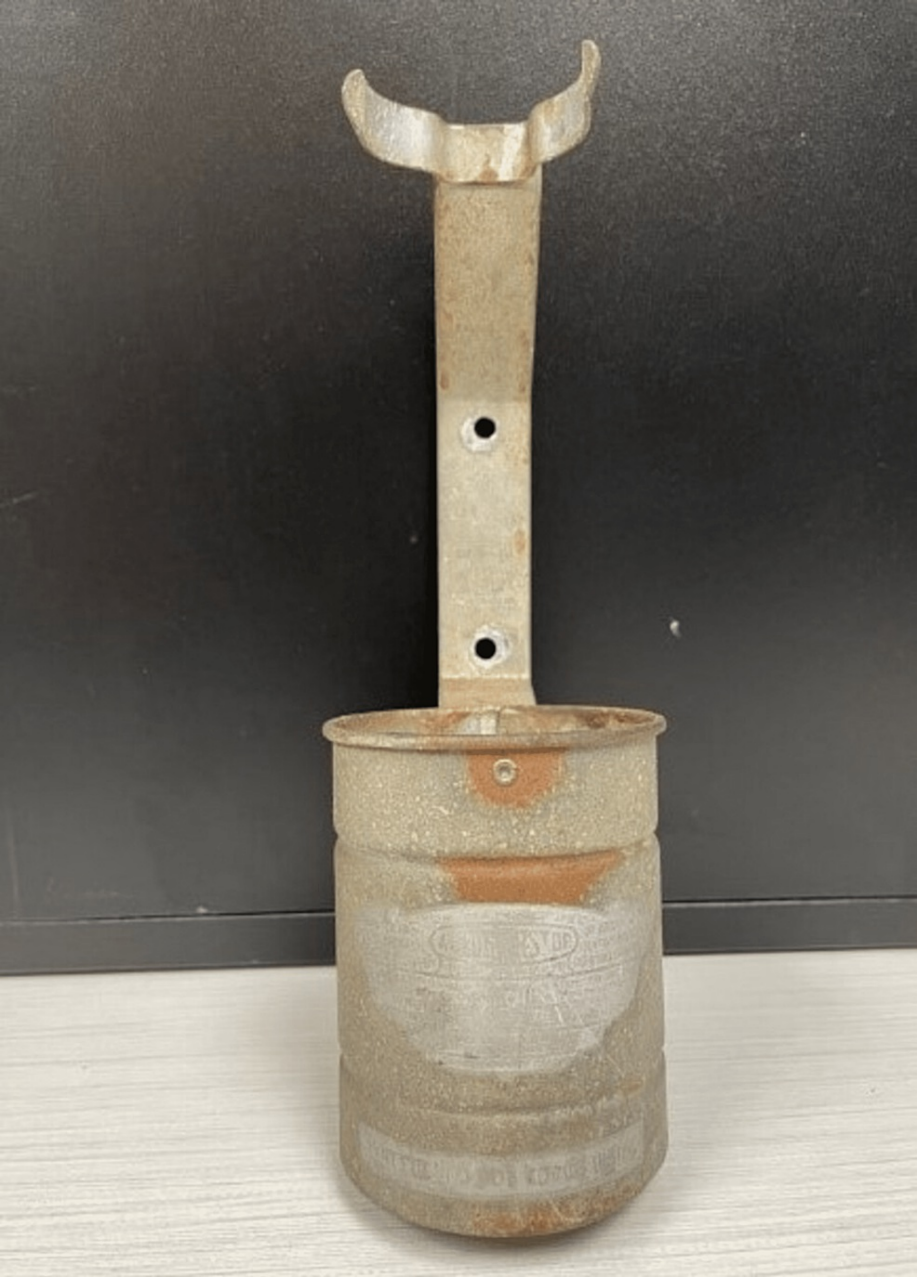
The antique firebomb stand photographed in the patients’ home.

The patient’s father (patient 2) was admitted one day prior with similar markedly elevated transaminases. History revealed that both patients had been exposed to CCl_4_ from an antique *firebomb* (Figure 1) that had shattered in the basement after being knocked off of an antique *firebomb* stand (Figure 2). Both patients had cleaned the debris without any personal protective equipment and later slept in the same contaminated room.

**Figure 3:**
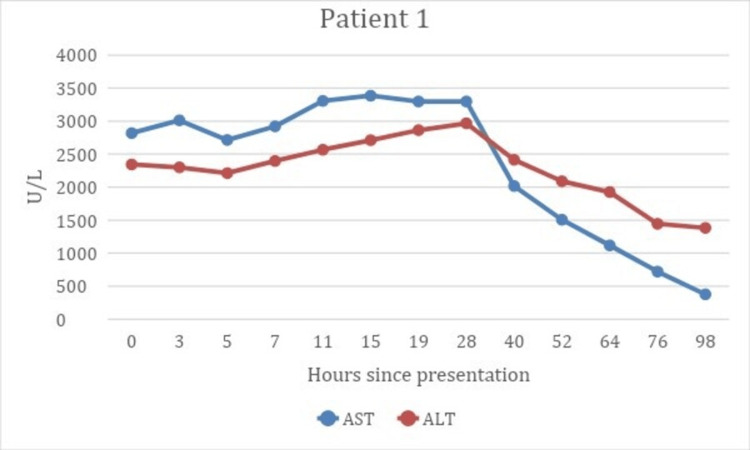
Patient 1’s AST and ALT values at intervals from presentation to discontinuation of N-acetylcysteine therapy. AST, aspartate aminotransferase; ALT, alanine aminotransferase

Toxicology was consulted and *N*-acetylcysteine (NAC) therapy was initiated. The patient received a 150 mg/kg loading dose over one hour, followed by a 50 mg/kg dose over four hours, and then 100 mg/kg every 16 hours for a total of 48 hours. The patient’s peak AST was 3,386 U/L and ALT was 2,968 U/L. NAC therapy was discontinued when the patient's AST was down trending and <1,000 U/L on two consecutive lab draws (Figure 3). Cimetidine 300 mg oral QID was also given for 48 hours. The patient had a complete resolution of symptoms and was discharged on hospital day (HD) five.

Patient 2

A 40-year-old male with a past medical history of colon cancer, diabetes mellitus type II, and alcohol use presented to the ED with one day of right upper quadrant and epigastric abdominal pain. Initial VS were BP as follows: BP, 125/88 mmHg; HR, 112 beats per minute; RR, 18 breaths per minute; T, 98.4 °F; and SpO_2_, 96% on room air. Pertinent physical examination findings included tenderness in the right upper quadrant without guarding or rebound and full-body jaundice.

**Figure 4:**
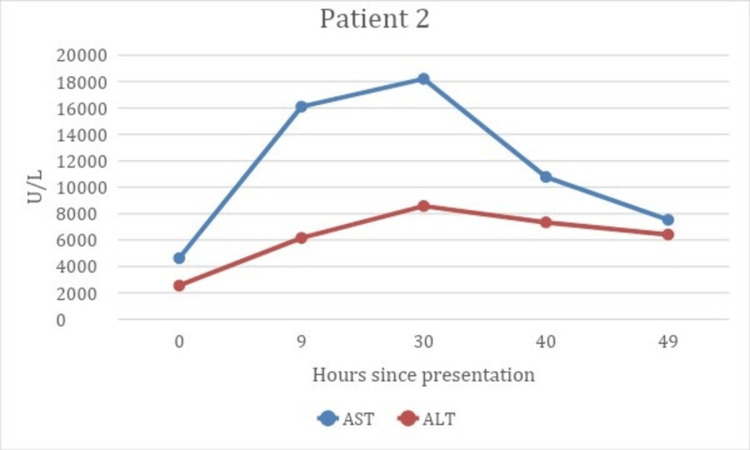
Patient 2’s AST and ALT values at intervals from presentation until transferred to a tertiary care center. AST, aspartate aminotransferase; ALT, alanine aminotransferase

Initial labs demonstrated markedly elevated transaminases with AST 4,625 U/L (<41) and ALT 2,550 U/L (<56), bilirubin 9.5 mg/dL (0.1-0.8), pH 7.31 (7.35-7.45), creatinine 1.08 mg/dL (0.53-1.30), PT 19.7 seconds (11-13.5), and INR 1.8 (0.8-1.1). Toxicology recommended NAC initiation at the same dosing strategy as patient 1. Throughout his stay, he developed worsening hepatic injury peaking at AST 18,203 U/L and ALT 8,572 U/L (Figure 4), worsening renal function with creatinine of 2.77 mg/dL, and worsening total bilirubin of 15.4 mg/dL. Abdominal imaging showed extensive hepatic steatosis with patent hepatic related vasculature, although specific findings of cirrhosis were not demonstrated in the liver. The patient was transferred to a tertiary care center with liver transplant capabilities. There he began to improve, but the patient left against medical advice on HD 5. The patient presented to the ED for an unrelated issue one month later, and AST was 176 U/L (<41) and ALT was 127 U/L (<56). 

## Discussion

CCl_4_ use in the United States declined when the Environmental Protection Agency restricted its commercial use in the mid-1960s, secondary to its severe toxicity. By 2009, 26 manufacturers worldwide produced CCl_4_, three in the United States [4]. Exposures to hydrocarbons like CCl_4_ continue to be a public health concern, specifically in populations such as unsupervised children with ingestions, workers prone to occupational exposures, and youth who abuse solvents by inhalation [5].

Hepatocellular necrosis is typically dose-dependent. Inhalation is the most common route of exposure, but oral ingestion or dermal absorption can also lead to toxicity. Metabolism of CCl_4_ occurs via cytochrome CYP2E1 into its toxic metabolite, trichloromethyl radical. This radical covalently binds to hepatocyte macromolecules causing lipid peroxidation and oxidative damage [5]. The centrilobular region of the liver (zone 3) is most often damaged given the high concentration of CYP2E1 enzymes. However, lethal exposures cause diffuse necrosis [4]. Nephrotoxicity occurs similarly via oxidative injury [6].

Acute exposure to inhaled CCl_4_ typically causes nonspecific symptoms, including dizziness, nausea, and vomiting. High-dose exposure can lead to respiratory depression, cardiac arrhythmias, coma, and death [2]. It is unclear how long CCl_4_ remains in the blood, but we suspect the blood levels in these cases were negative, given the delay in presentation (>60 hours) from exposure. In the setting of known exposure and lack of other identified causes, both patients’ hepatic injuries can be attributed to CCl_4_ exposure. Acute organ dysfunction typically occurs one to four days after initial exposure, as seen in our patients. Acute hepatic injury presents with tender hepatomegaly, elevated hepatic enzymes, decreased albumin and fibrinogen, and elevated PT/INR [4].

Treatment of CCl_4_ toxicity is aimed at preventing further damage. Initial management is decontamination via skin washing, eye irritation, and gastric lavage if indicated due to ingestion exposure. Dialysis and hyperbaric oxygen can be considered in select patient populations [4]. Hyperbaric oxygen is controversial secondary to the risk of additional free radical damage [7]. However, it was shown to be protective against hepatotoxicity in rat models [8]. 

NAC, an antioxidant that can scavenge reactive oxygen species, can decrease hepatic damage. NAC is hydrolyzed into cysteine, which produces glutathione and enhances glutathione-*S*-transferase activity. This process protects against oxidative stress and promotes detoxification [9]. Research shows that NAC therapy in CCl_4_ toxicity reduces serum liver marker enzymes and promotes liver healing [10].

Cimetidine is an H_2_ receptor antagonist, which may also be useful in the treatment of CCl_4_ hepatotoxicity by impairing cytochrome P450, the main enzyme responsible for metabolizing CCl_4_ to trichloromethyl radical. Cimetidine also promotes the stimulation of regenerative processes acting on DNA synthesis [11].

## Conclusions

CCl_4_ toxicity should be considered in patients with elevated liver function tests and exposure to an unknown substance. Acute exposure to CCl_4_ in a high concentration can potentially result in hepatocellular necrosis. Early recognition and treatment with NAC with the co-administration of cimetidine if indicated is crucial in treating liver damage and preventing any damage that may have been caused. CCl_4_ toxicity is rare and sparsely reported in current literature but should be maintained in the differential of acute hepatitis. Exposure to antiquated xenobiotics like CCl_4_ is rare but important for the public to be aware of due to their profound toxicity.
